# Feasibility of high-dose dobutamine stress SSFP cine MRI at 3 Tesla with patient adaptive local RF shimming using dual-source RF transmission: initial results

**DOI:** 10.1186/1532-429X-13-S1-P101

**Published:** 2011-02-02

**Authors:** Katharina A Strach, Andreas Müller, Marc Kouwenhoven, Ralf Clauberg, Claas P Naehle, Jürgen Gieseke, Hans H Schild, Daniel Thomas

**Affiliations:** 1 Department of Radiology and Nuclearmedicin, University of Magdeburg, Magdeburg, Germany; 2 Department of Radiology, University of Bonn, Bonn, Germany; 3 Philips Healthcare, Best, Netherlands; 4 Philips Healthcare, Hamburg, Germany

## Introduction

Cine SSFP sequences have become the gold standard for assessment of myocardial function at 1.5T. However, cine imaging at higher-field strengths is hampered by SAR limitations, the increased inhomogeneity of the B1 (RF) and B0 field and the high sensitivity of SSFP sequences to off-resonance artefacts. The effect is even more pronounced in HDDS studies, which are associated with a significant increase in heart rate and consequently more complex and faster blood flow. Previously, spoiled gradient-echo sequences have been proposed as an alternative for stress studies at 3T. Recently, the introduction of a dual-source RF transmission system with patient-adaptive local RF-shimming has lead to a significant improvement of image quality of SSFP imaging at 3T during resting conditions and may thus allow for reliable imaging of cardiac function even at high heart rates.

## Purpose

To investigate the feasibility of high-dose dobutamine stress (HDDS) imaging using SSFP sequences at 3T employing patient-adaptive local RF-shimming using a dual-source RF transmission system.

## Methods

8 Patients with suspected CAD underwent a HDDS protocol on a 3T MRI scanner (Achieva 3.0T-TX, Philips Healthcare, Best, Netherlands), equipped with a dual-source RF transmission system. SSFP cine sequences at every stress level were performed in the short (SA), vertical (VLA) and horizontal long axis (HLA) using patient-adaptive local RF-shimming (RF-S) and compared to cine images acquired with identical sequence parameters at rest and maximal stress, without additional shimming. Overall image quality was evaluated on a 4-point grading scale (4: excellent, 1: non-diagnostic) and number of non-diagnostic segments assessed. Contrast between interventricular septum and blood pool was calculated [(SI_B_-SI_M_)/(SI_B_+SI_M_)].

## Results

All HDDS studies were completed successfully. RF-S lead to a significant improvement of overall image quality at stress (SA 3.62+/-0.52 vs. 2.13+/-0.64; VLA 2.88+/-0.35 vs. 1.75+/-0.46; HLA 3.13+/-0.64 vs. 1.5+/-0.76; p<0.05) and in both long axis at rest (VLA 3.38+/-0.52 vs. 2.88+/-0.35; HLA 3.63+/-0.52 vs. 2.75+/-0.71; p<0.05) compared to sequences without RF-S. The amount of non-diagnostic segments was significantly reduced when using RF-S at rest (0 vs. 20, p<0.05) and maximum stress (4 vs. 77, p0.05) with RF-S, the difference was not statistically relevant.

## Conclusion

Patient-adaptive local RF-shimming using a dual-source RF transmission MRI system allows for reliable SSFP imaging in a clinical high-dose dobutamine stress protocol. RF-S significantly improves image quality and reduces the number of non-diagnostic myocardial segments.

